# Diclofenac Enhances Docosahexaenoic Acid-Induced Apoptosis in Vitro in Lung Cancer Cells

**DOI:** 10.3390/cancers12092683

**Published:** 2020-09-20

**Authors:** Rosemary A. Poku, Kylee J. Jones, Megan Van Baren, Jamie K. Alan, Felix Amissah

**Affiliations:** 1 Department of Foundational Sciences, College of Medicine, Central Michigan University, Warriner Hall, 319, Mt Pleasant, MI 48859, USA; poku1r@cmich.edu; 2 Department of Pharmaceutical Sciences, Ferris State University, College of Pharmacy, 220 Ferris Dr, Big Rapids, MI 49307, USA; redi5@ferris.edu (K.J.J.); vanbarm@ferris.edu (M.V.B.); 3 Department of Pharmacology and Toxicology, Michigan State University, East Lansing, MI 48824, USA; alanjami@msu.edu

**Keywords:** polyunsaturated fatty acids, docosahexaenoic acid, K-Ras, cyclooxygenase, non-steroidal anti-inflammatory drugs, diclofenac, lung cancer, nanostring

## Abstract

**Simple Summary:**

Polyunsaturated fatty acids (PUFAs) and non-steroidal anti-inflammatory drugs (NSAIDs) have limited anticancer capacities when used alone. We examined whether combining NSAIDs with docosahexaenoic (DHA) would increase their anticancer activity on lung cancer cell lines. Our results indicate that combining DHA and NSAIDs increased their anticancer activities by altering the expression of critical proteins in the RAS/MEK/ERK and PI3K/Akt pathways. The data suggest that DHA combined with low dose diclofenac provides more significant anticancer potential, which can be further developed for chemoprevention and adjunct therapy in lung cancer.

**Abstract:**

Polyunsaturated fatty acids (PUFAs) and non-steroidal anti-inflammatory drugs (NSAIDs) show anticancer activities through diverse molecular mechanisms. However, the anticancer capacities of either PUFAs or NSAIDs alone is limited. We examined whether combining NSAIDs with docosahexaenoic (DHA), commonly derived from fish oils, would possibly synergize their anticancer activity. We determined the viability of lung cancer cell lines (NCI-H1573, A549, NCI-H1299, and NCI-H1975) after exposure to DHA and various NSAIDs. We further conducted cell apoptosis assays and analyzed apoptosis-associated proteins and some key proteins in the RAS/MEK/ERK and PI3K/Akt pathways using western blot analysis. We also determined the impact of the treatment on the expression of inducible cancer-related genes using nCounter PanCancer Pathways gene expression analysis. The results showed that the combination of DHA and NSAIDs increased suppression of cell viability in all the lung cancer cell lines tested compared to each of the compounds used alone, with diclofenac being the most potent NSAID tested. This synergistic effect is especially significant in A549 and NCI-H1573 cells. The combination treatment was more effective at inhibiting clonogenic cell growth and anchorage-independent growth in soft agar, inducing caspase-dependent apoptosis, and altering expression of critical proteins in the RAS/MEK/ERK and PI3K/Akt pathways. The data from this study demonstrate that DHA combined with low dose diclofenac provides greater anticancer potential, which can be further developed for chemoprevention and adjunct therapy in lung cancer.

## 1. Introduction

Lung cancer continues to pose a serious health problem in the US as the second most commonly diagnosed cancer with an estimated 228,820 new diagnoses and 135,720 deaths likely to occur in 2020. Thus, lung cancer is projected to account for one-quarter of all cancer-related deaths. With an overall 5-year survival rate of only 19% for all lung cancer stages combined [1], there is a need for effective preventive and therapeutic strategies to combat this disease. Chemoprevention, which involves the use of dietary or pharmaceutical interventions to slow or reverse the progression of cancer, is important for patients with an elevated risk of cancer [2,3]. Notable among the compounds with promising potential for inhibiting the progression of cancer are the cyclooxygenase (COX) inhibitors and omega-3 (ω-3) fish oil [4,5,6]. These two classes of medications, mostly available “over-the-counter” have been under extensive investigation in the past few decades for their beneficial health effects.

Cyclooxygenases are the main enzymes involved in the conversion of polyunsaturated fatty acids (PUFAs) to prostaglandins (PGs) and other eicosanoids [7,8]. Overexpression of Cyclooxygenase-2 (COX-2), a key mediator of inflammation, promotes transformed and invasive phenotypes with increased cell proliferation, motility, invasion, angiogenesis, and resistance to apoptosis [9]. COX-2 remains an important target for colorectal cancers, and more recently, lung cancer therapy and prevention because approximately 70% of lung adenocarcinomas overexpress COX-2 [10]. Targeting COX enzymes for cancer prevention and therapy is supported by several clinical and epidemiological studies [11,12,13]. Pharmacological inhibition with non-steroidal anti-inflammatory drugs (NSAIDs) or genetic deletion of COX-2 significantly diminishes tumor formation in several cancer models [14,15,16]. Evidence from recent studies also implicates COX-1 in the chemopreventive roles of NSAIDs [4,6,17,18,19]. COX-dependent mechanisms involving decreased production of pro-oncogenic PGE_2_ has been reported as part of the anticancer effects of NSAIDs [8,12,20]. However, several other studies have suggested COX-independent mechanisms for NSAIDs, which involve modulation of NFκB, TGF-β, and Wnt/β-catenin signaling, interference with the Ras-Raf-MEK-ERK signaling cascade, the PI3K/Akt/MAPK signaling axis and/or activation of PPARs [11,21,22,23].

Similarly, numerous epidemiological studies strongly support the anticancer effects of long-chain polyunsaturated fatty acids (PUFAs), especially the ω-3 docosahexaenoic acid (DHA) and eicosapentaenoic acid (EPA) commonly found in fish oils. The ω-3 PUFAs, mainly present in fish oil, form an important part of the human diet. Their chemopreventive effects have been demonstrated both in vitro, and in vivo using several models, indicating that ω-3 PUFAs significantly inhibit tumor growth, suppress cell viability, and induce apoptosis in various cancer cells [5,24,25,26]. Among the mechanisms proposed for their anticancer effects include modulation of survival signaling pathways such as Wnt/β-catenin, MAPK/Erk, PI3K/Akt/mTOR, JAK-STAT, and NF-κB [5,24,27,28,29,30]. While it is possible to experience enhanced anti-cancer effects by combining NSAIDs with PUFAs, this prospect is yet to be fully explored. In this study, we examine the effects of the combination of ω-3 PUFAs and NSAIDs on a panel of lung cancer cells. We also explored the possible mechanism of action involved in their anticancer effects. We focused on DHA in the current study due to its superior antitumor potency as well as the recent attention it has received for its anticancer properties among ω-3 PUFAs [31,32,33]. We report here that diclofenac exhibits the most potent effects compared to the other NSAIDs used and has a positive synergistic cytotoxic interaction with DHA. Exploration of this novel interaction shows that the combination interferes with Ras/MEK/ERK and PI3K/Akt signaling pathways.

## 2. Results

### 2.1. Co-Treatment of Lung Cancer Cells with NSAIDs and DHA Enhanced Cytotoxicity

**Figure 1:**
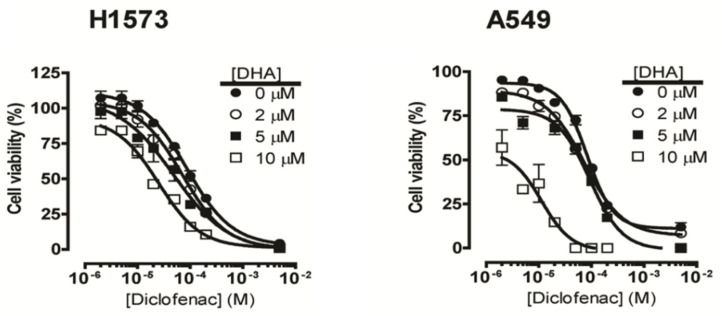
Suppression of cell viability by co-treatment of DHA and diclofenac in NCI-H1573 and A549 human lung cancer cells. Cultured cells were seeded in 96-well plates seeded at a density of 1 × 10^4^ were exposed to diclofenac (0–500 µM) alone or with DHA (● 0 µM, ○ 2 µM, ■ 5 µM or □ 10 µM) for 48 h. Cell viabilities were determined after the final treatment by fluorescence using the resazurin reduction assay. Each point represents the mean ± SEM relative to the control untreated cells.

**Figure 2:**
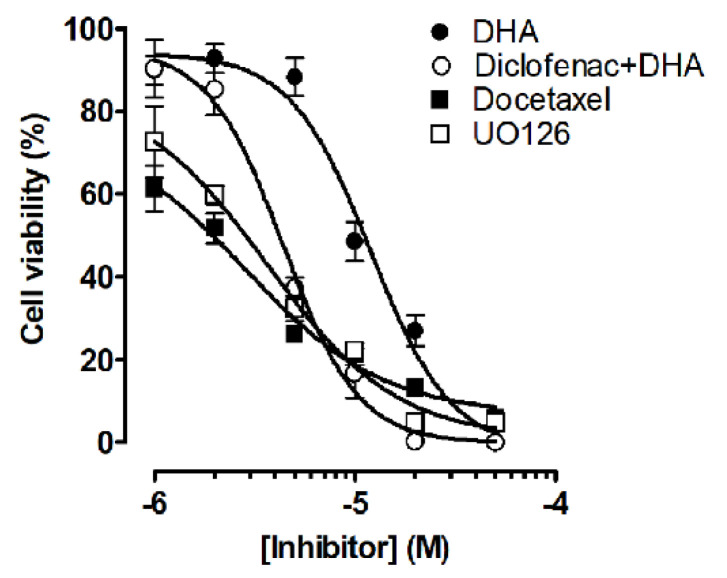
Comparing the effectiveness of co-treatment of DHA and diclofenac with docetaxel and UO126 on the viability A549 human lung cancer cells. The effects DHA (●) alone or with 25 µM of diclofenac (○) were compared to docetaxel (■) and UO126 (□) for 48 h. Cell viabilities were determined after the final treatment by fluorescence using the resazurin reduction assay. The IC_50_s were 11.1 ± 1.32 µM, 4.38 ± 0.52 µM, 2.82 ± 1.42 µM, and 3.61 ± 1.2 µM for DHA alone, DHA with diclofenac, docetaxel, and UO126, respectively. Each point represents the mean ± SEM relative to the control untreated cells.

To determine the anticancer potential of the combination of NSAIDs and DHA, we co-treated NCI-H1573 cells with increasing concentrations of DHA (1–50 μM) and various NSAIDs (10–100 μM) for 48 h. These specific NSAIDs were selected to represent some of the major chemical classes (acetic acid derivatives—indomethacin and diclofenac; propionic acid derivative—naproxen; enolic acid derivative—piroxicam) as well as a wide range of relative cyclooxygenase selectivity [34,35]. The cell viability results showed that treatment with DHA alone resulted in concentration-dependent growth inhibition. For example, in the NCI-H1573 cells, an IC_50_ of 9.5 ± 1.3 μM was noted for DHA alone (Table 1). However, co-treatment of DHA with either diclofenac or indomethacin at 25, 50 and 100 μM markedly enhanced the cytotoxicity of DHA, as indicated by the significant decrease in the IC_50_ values (Table 1), and the leftward shift of the concentration-response curves with suppressed maximal cell viability. Likewise, co-treatment with diclofenac in A549 cells decreased the IC_50_ of DHA from 9.5 ± 1.1 to 3.7 ± 0.4 in the presence of 100 μM of diclofenac. Piroxicam and naproxen were found to be less effective in promoting the cytotoxic effects of DHA. Diclofenac was found to be more effective at enhancing the cytotoxic effect of DHA in all the cell lines used. We further established concentration-response curves for diclofenac alone and with DHA. Similarly, diclofenac inhibited the viability of NCI-H1573 cells in a concentration-dependent manner with an IC_50_ of 87.5 ± 9.6 μM, while the IC_50_ values of co-treatment with 2, 5, and 10 μM of DHA decreased to 71.4 ± 9.0 μM, 48.3 ± 1.4 μM, and 25.0 ± 3.4 μM, respectively (Figure 1, Table 2). The IC_50_ of diclofenac on A549 cell viability was 92.8 ± 9.9 μM, while co-treatment with 2, 5, and 10 μM of DHA decreased the IC_50_ values to 83.1 ± 4.3 μM, 73.5 ± 6.5 μM, and 11.3 ± 3.5 μM, respectively, indicating enhanced cytotoxicity in the co-treatment groups. Because of its superior performance, we employed diclofenac (25 μM) in the co-treatment with DHA for subsequent experiments. Although similar effects were observed in NCI-H1573, A549, NCI-H1299, and NCI-H1975 when co-treated with DHA and diclofenac (Table 1), the inhibitory effects were more pronounced in the NCI-H1573 and A549 cells. These two cell lines, NCI-H1573 and A549 are known to harbor activating KRAS (G12A) and KRAS (G12S) mutations, respectively. In addition, the cytotoxic effect of DHA and diclofenac (25 μM) on A549 cells was compared to docetaxel, a microtubule inhibitor used in lung cancer chemotherapy and UO126, a MEK inhibitor that acts downstream in the Ras signaling pathway. Co-treatment of A549 cells with DHA and diclofenac (25 μM) was only slightly less potent at reducing the A549 cell viability compared to docetaxel and UO126 (Figure 2). The IC_50_s were 11.1 ± 1.3 µM, 4.4 ± 0.5 µM, 2.8 ± 1.4 µM, and 3.6 ± 1.2 µM for DHA alone, DHA with diclofenac, docetaxel, and UO126, respectively.

**Figure 3:**
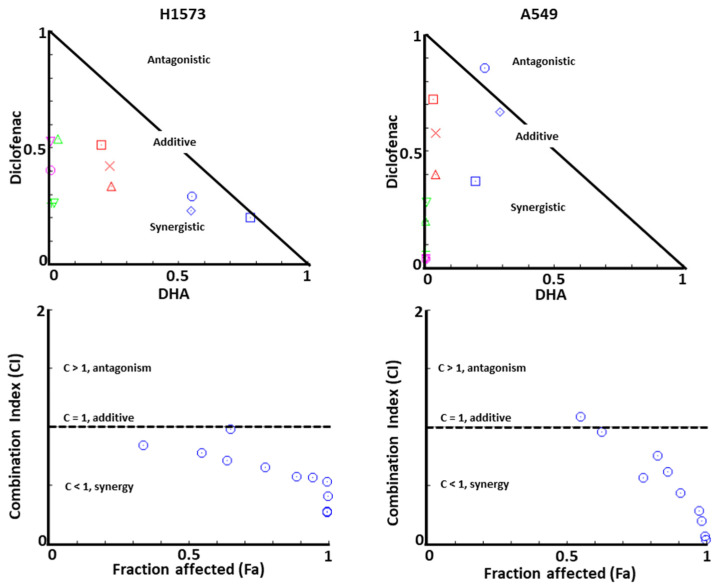
Normalized isobologram of combined treatment of DHA and diclofenac on NCI-H1573 and A549 human lung cancer cells. The combination index for DHA and diclofenac on NCI-H1573 cells were analyzed using CompuSyn software. The different colored symbols represent fractional activities for different concentrations of DHA and diclofenac used in the experiment. CI < 1, = 1, and > 1 indicates synergistic, additive, and antagonistic effects, respectively.

As shown in Figure 3, the CI values of DHA and diclofenac in combination were mostly less than one, suggesting that the growth inhibitory effect of these compounds in combination was mostly synergistic rather than additive or antagonistic in NCI-H1573 cells. Diclofenac at 25 μM and DHA at 5 μM used alone induced slight cytotoxicity on NCI-H1573 cells with fractional effects of 0.18 and 0.04, respectively. However, the combination treatment of diclofenac (25 μM) and DHA (5 μM) showed marked synergism with a fractional effect of 0.64 (CI = 0.72).

### 2.2. Co-Treatment of Lung Cancer Cells with DHA and Diclofenac Decreased Clonogenic Cell Survival

**Figure 4:**
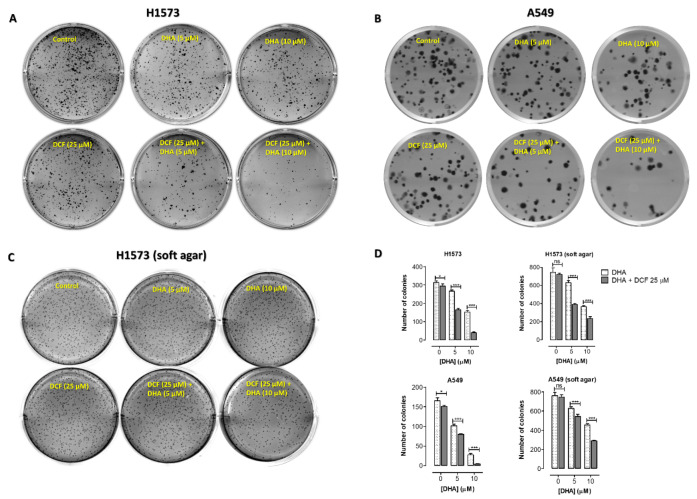
Clonogenic cell survival and anchorage-independent cell growth of NCI-H1573 and A549 cells in response to co-treatment with DHA and diclofenac (DCF). Representative images of cells treated with DHA and diclofenac are shown. (**A,B**) For clonogenic cell survival assays, cultured cells were pre-treated with DHA (0–10 μM) and DCF (25 μM) for 48 h. Cells were then trypsinized, washed, counted and incubated for a further 10–14 days at 37 °C after which the clonogenic survival was determined. (**C**) For the anchorage independent growth (soft agar) assay, counted cells were plated in soft agar and treated weekly as described in the methods for 21 days. (**D**) The number of colonies formed were counted, and the results are expressed as the means (± SEM, *n* = 4) relative to the DHA alone treatments. Significance (* *p* < 0.05; *** *p* < 0.001) was determined by Student’s *t*-test.

We further compared the ability of NSCLCs to survive, grow and form colonies after exposure to DHA and diclofenac alone or in combination. We observed that DHA alone induced concentration-dependent inhibition of cell survival and colony formation in both NCI-H1573 and A549 cells, and this inhibitory effect was significantly increased with the addition of diclofenac. As shown in Figure 4, prior exposure of A549 cells to DHA (5 μM) alone or with diclofenac (25 μM) for 48 h, followed by re-plating of the cells and allowing them to grow for 12–14 days revealed a reduction in survival by 38.4 ± 2.3% and 51.7 ± 0.9% (*p* < 0.001), respectively. Exposure to DHA (10 μM) alone or in combination with diclofenac (25 μM) also inhibited the colony formation by 83.5 ± 2.3 % and 97.4 ± 0.5% (*p* < 0.001), respectively. These results indicate that treatment with DHA had a concentration-dependent effect on colony formation, which is amplified when the cells are co-treated with diclofenac.

In addition, anchorage-independent growth in soft agar, which is correlated with tumor progression, was significantly reduced in NCI-H1573 and A549 cells exposed to DHA (10 μM) in combination with diclofenac (25 μM) (Figure 4C,D). These results further indicate that co-treatment with DHA and diclofenac inhibited tumorigenicity of NCI-H1573 and A549 cells.

### 2.3. Co-Treatment of Lung Cancer Cells with DHA and Diclofenac Induces Apoptosis

**Figure 5:**
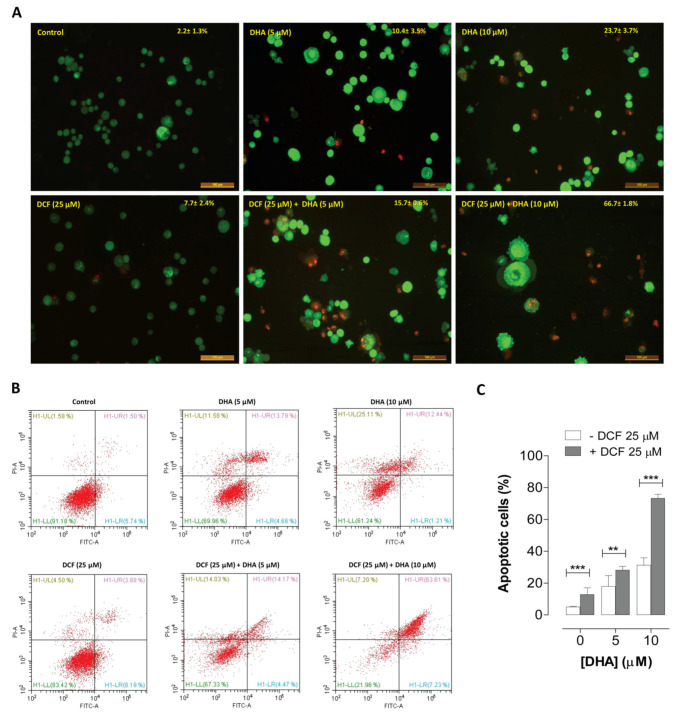
Diclofenac (DCF) increased DHA-induced apoptosis in lung cancer A549 cells. (**A**). Cells treated with DHA and DCF for 48 h were analyzed for apoptosis using acridine orange and ethidium bromide (AO/EB, 10 μg/ml) as described in the methods. Stained cells were visualized with a Leica DM5000B fluorescent microscope (magnification ×20). When stained with AO/EB, live cells with normal nuclei appear green while apoptotic cells (**A**) with condensed or fragmented chromatin in the nuclei appear orange. (**B**) Representative scatter plots illustrating Annexin V-FITC /PI staining of cells after treatment of A549 cells with DHA (0–10 μM) and DCF (25 μM) for 48 h. Annexin-V vs. PI plots were generated via flow cytometry cell sorting technology. The percentage of the apoptotic cell death increased in cells co-treated with DHA and DCF. (**C**). Co-treatment of A549 cells with DHA and DCF resulted in a dramatic increase in the percentage of apoptotic cells (early and late apoptotic cells). Data were expressed as means (± SEM, *n* = 3). ** *p* < 0.01; *** *p* < 0.001 indicate significant differences compared to treatments with DHA alone.

Cytotoxicity induced by compounds may result from necrosis, which is pro-inflammatory, or apoptosis (programmed cell death) characterized by membrane blebbing, cellular shrinkage, nuclear condensation and fragmentations, and formation of apoptotic bodies [36]. For an anticancer effect, it is desirable to induce apoptosis because it is not associated with significant inflammation. To determine whether the cytotoxic effect of DHA and diclofenac is associated with an increase in apoptosis, the morphological changes and the mode of cell death induced by DHA and diclofenac were determined by staining A549 cells with acridine orange/ethidium bromide (AO/EB) after a 48 h incubation with the compounds. AO permeates live cells and stains the nuclei, which appear green. EB permeates only the cells with compromised plasma membrane integrity and stains the nuclei red. As shown in Figure 5A, no significant apoptosis was detected in the vehicle-treated control group (2.2 ± 1.3%). Very low levels of apoptosis were detected in the A549 cells treated with diclofenac (7.7 ± 2.4%) and 5 μM of DHA alone (10.4 ± 3.5%). An increase in apoptosis was detected in A549 cells co-treated with 10 μM of DHA and diclofenac (66.7 ± 1.8%) compared to the treatment with 10 μM of DHA alone (23.7 ± 3.7%), which was signified by an increase in cells with marked red-stained nuclei (due to EB uptake following the loss of cytoplasmic membrane integrity).

These results were further confirmed using an Annexin V/propidium iodide staining test to assess apoptosis. As shown in Figure 5B,C, we observed significantly higher apoptotic rates in A549 cells co-treated with DHA and diclofenac compared to the groups of single treatments. These data suggest that combining DHA and diclofenac induced a significant increase in apoptosis in lung cancer cells compared to treatment with DHA alone.

**Figure 6:**
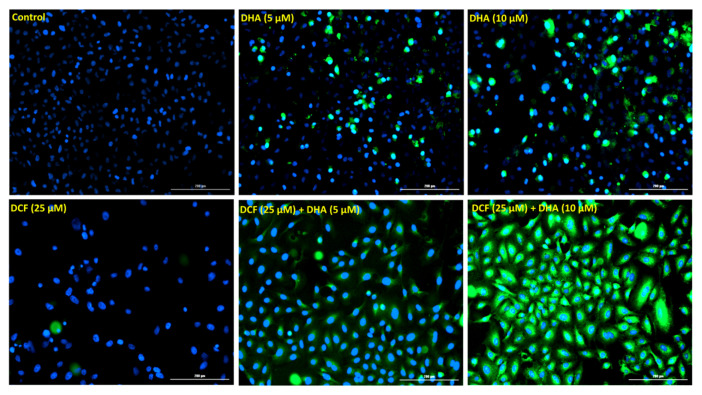
Co-treatment with DHA and DCF induced caspase 3/7 activation in A549 cells. Caspase 3/7 activation was observed after treating A549 cells with DHA and DCF for 48 h and then reacting with the fluorescent caspase 3/7 irreversible inhibitor (green FLICA). Images were taken with the Cytation 1 Cell Imaging Multi-Mode Reader (magnification ×10).

The observed characteristics of apoptosis induced in the A549 cells above can be attributed at least in part to a series of activation of the caspase family of cysteine proteases, which culminates in the activation of executioner caspases, leading to mass proteolysis. Therefore, we further investigated the involvement of executioner caspases 3 and 7 in the apoptotic effect of DHA and diclofenac in A549 cells. Results from the CaspaTag^TM^ Caspase-3/7 in situ assay indicated that the co-treatment with DHA and diclofenac was more effective at activating caspases 3/7 in A549 cells compared to treatment with either compound individually (Figure 6). A549 cells co-treated with DHA and diclofenac showed prominent activation of caspase 3/7, as indicated by intense green fluorescence in the cells compared to the control cells. Treatment with DHA or diclofenac alone was less effective at activating the caspases 3/7, as shown in Figure 5A.

**Figure 7:**
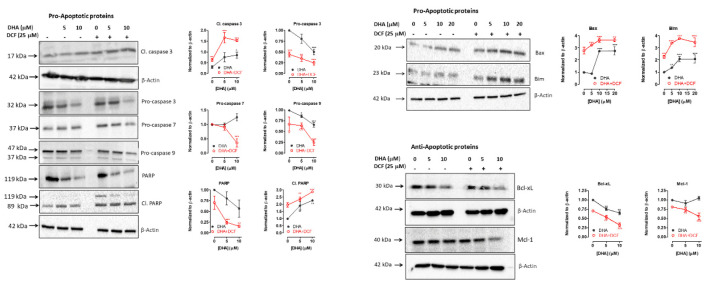
Diclofenac (DCF) and DHA induce the expression of pro-apoptotic proteins. Western blot analysis showing the expression of pro-apoptotic and anti-apoptotic proteins in A549 cells after treatment with DHA (0–10 μM) and DCF (25 μM) for 48 h. The blots were quantified using Biorad Image Lab software and analyzed using one-way analysis of variance followed by Bonferroni’s multiple comparison test. Results are from three independent experiments (mean ± SEM, *n* = 3). * *p* < 0.05, ** *p* < 0.01, and *** *p* < 0.01 compared to control. Detailed information about western blot can be found at Figure S1.

In addition, enhanced induction of apoptosis by the combination treatment was further evidenced by western blot analyses showing increased expression levels of cleaved caspase 3, cleaved PARP, and pro-apoptotic proteins (Bax and Bim) as shown in Figure 7. We also detected a corresponding decrease in expression of PARP, procaspase-3, procaspase-7, procaspase-9, and anti-apoptotic proteins (Bcl-xL and Mcl-1). These results demonstrate that the apoptotic cell death induced by co-treatment with DHA and diclofenac can be attributed at least in part to the induction of caspase-activation in lung cancer cells.

### 2.4. Co-Treatment of Lung Cancer Cells with DHA and Diclofenac Altered Expression of Cancer-Related Proteins in A549 and NCI-H1573 Cells

We further explored the anticancer potential of co-treatment with DHA and diclofenac on A549 and NCI-H1573 cells using the nCounter PanCancer Pathways gene expression analysis. This assay determines the expression of a broad panel of inducible cancer-related genes categorized in major oncogenic pathways using RNA profiling technology that employs molecularly bar-coded fluorescent probes (NanoString Technologies, WA, USA). We found that treatment with DHA (5 and 10 μM) altered the expression of several genes in both A549 and NCI-H1573 lung cancer cell lines. Concomitant treatment with DHA and diclofenac further increased the extent to which the expression of those same genes was altered. When the results for co-treatment with DHA (10 μM) and diclofenac (25 μM) were used in the subsequent analysis and a fold change of ≥ ± 2 were considered significant, a total of 88 genes in the A549 cells and 97 genes in the NCI-H1573 cells were significantly altered. Expression patterns of the top 60 genes that were altered in both A549 and NCI-H1573 cell lines were further organized according to their associated signaling pathways as described by the nCounter PanCancer Pathways code set into Ras signaling, MAP kinase signaling, PI3K/Akt signaling, driver genes, cell cycle/apoptosis regulation, DNA damage control, APC/Wnt signaling, and transcriptional regulation (Table S1). Co-treatment with DHA and diclofenac effectively induced the expression of pro-apoptotic and cell cycle arrest genes such as growth arrest and DNA damage-inducible alpha (GADD45A) and tumor necrosis factor, alpha-induced protein 3 (TNFAIP3). However, anti-apoptotic/cell proliferation genes and transcription factors such as E2F1, proliferating cell nuclear antigen (PCNA), cyclin-A2, -B1, and E2 were downregulated.

**Figure 8:**
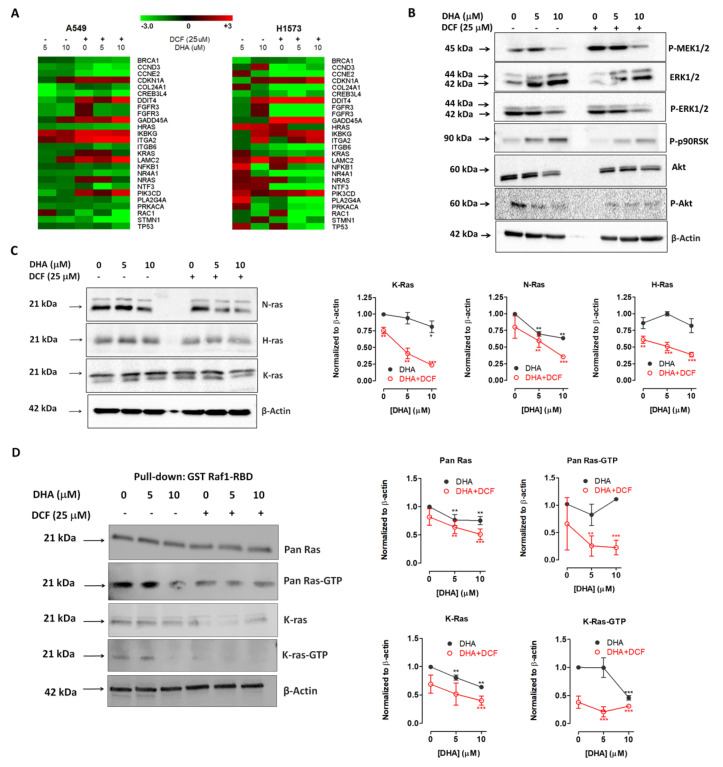
Co-treatment with diclofenac (DCF) and DHA alters the expression of proteins in the Ras/MEK/ERK and PI3K/AKT pathways. (**A**). Heatmap illustrating changes in mRNA expression for Ras/MAPK and PI3K/AKT genes altered in A549 and NCI-H1573 cells exposed to DHA (5 and 10 μM) alone or with DCF (25 μM) for 48 h as determined by the nCounter PanCancer Pathways gene expression analysis. The heat map of gene expression with fold changes of ≥ ± 2 with key roles in the Ras/MEK/ERK and PI3K/AKT pathways was generated using the MultiExperiment Viewer (MeV v4.9.0, http://www.tm4.org) (red indicates upregulated genes; green indicates downregulated genes). Western blot analysis showing the expression of (**B**). phospho-MEK1/2, phospho-p44/42 MAPK (Erk1/2), phospho-p90RSK phospho-Akt, and Akt (pan); (**C**). K-Ras, N-Ras, and H-Ras in A549 cells after treatment with DHA (0–10 μM) and DCF (25 μM) for 48 h. (**D**). Western blot analysis of GTP-bound K-Ras and pan Ras following pull-down with the GST-Raf1-RBD fusion protein, which binds only active Ras, indicated decreased Ras activity after co-treatment of A549 cells with DCF (25 μM) and DHA (5 and 10 μM) for 48 h. Normalized densitometric data was used to generate the graphs shown in GraphPad Prism 5.0 (La Jolla, CA, USA). Statistical significance (* *p* < 0.05; ** *p* < 0.01, *** *p* < 0.001) was determined by comparing the mean of each treatment group to untreated control using a 1-way ANOVA and post-hoc Dunnett’s test. Detailed information about western blot can be found at Figure S2.

Also, several genes involved in the DNA damage control (POLE2, RAD51, UBE2T) and transcriptional regulation (HIST1H3B, CDKN2C) were downregulated (Table S1). Key driver genes such as K-RAS, HRAS, NRAS, RAC1, and RHOA, which play important roles in Ras signaling, MAP kinase signaling, and PI3K/Akt signaling pathways were downregulated (Figure 8A).

### 2.5. Co-Treatment of Lung Cancer Cells with DHA and Diclofenac Inhibits the Ras/MEK/ERK and PI3K/AKT Signaling Pathways

The PI3K/AKT and Ras/MEK/ERK pathways are frequently dysregulated in many human tumors as a result of activating mutations in Ras or loss of negative regulatory proteins such as Phosphatase and tensin homolog (PTEN) and other proteins in the pathway. Mutant Ras oncogenic proteins are critical drivers across many cancers, including NSCLCs [37]. Based on our results from the gene expression data and reports from previous studies on the pathways commonly modulated by DHA and diclofenac when used alone [15,30], we investigated the impact of co-treatment with DHA and diclofenac on the PI3k/Akt and Ras/MEK/ERK pathways in A549 cells. We analyzed lysates generated from A549 cells following 48 h treatment with the DHA (0–10 μM) and diclofenac (25 μM). Exposure of the cells to DHA (10 μM) and diclofenac (25 μM) suppressed the protein levels of AKT, phospho-AKT, phospho-MEK1/2, phospho-p44/42 MAPK (p-ERK), and phospho-p90RSK (Figure 8B). However, p44/42 MAPK (Erk1/2) expression was relatively increased. We also observed a significant decrease in the levels of the Ras GTPase isoforms (K-Ras, H-Ras, and N-Ras) after 48 h exposure to the co-treatment with 10 μM of DHA and 25 μM of diclofenac (Figure 8C). We chose to examine the expression of these Ras isoforms because we found them to be downregulated in the nanostring assay and also, because they are frequently mutated in lung cancers, especially K-Ras [38]. A pull-down assay detected decreased activity of pan-Ras as well as K-Ras after co-treatment with DHA and diclofenac (Figure 8D). Taken together, we provide evidence that co-treatment with DHA (10 μM) and diclofenac (25 μM) effectively interfered with the relative amounts and activity of key proteins in the PI3K/AKT and Ras/MEK/ERK pathways to render cells susceptible to apoptosis.

## 3. Discussion

Omega-3 PUFAs, such as DHA, and NSAIDs, such as diclofenac, exhibit remarkable anticancer activities via diverse molecular mechanisms [11,30,39]. However, their anticancer capabilities are limited due to either the high doses required for their anticancer effect and the resultant potential adverse effects for diclofenac or limited clinical data regarding efficacy for DHA. In this study, we tested the combined anticancer effects of DHA and selected NSAIDs on NSCLC cell lines. We speculated that combination treatment with DHA and various NSAIDs could target different pathways simultaneously and therefore exert synergistic anticancer effects. The data demonstrated that co-treatment with DHA and NSAIDs, specifically diclofenac, exerted a synergistic inhibitory effect on the NSCLC cell line viability by inducing apoptotic cell death.

Inflammatory processes have been implicated in several human cancers and there are numerous reports that COX-2 overexpression and prostaglandins play a critical role in the development and progression of tumors [40]. Indeed, targeting cyclooxygenase-2 has been at the center of anti-cancer drug development. However, despite three decades of epidemiological, clinical, and experimental studies providing strong evidence of anticancer activity, the use of NSAIDs such as diclofenac for cancer chemoprevention is not recommended due to the potential risk of gastrointestinal, renal, and cardiovascular side effects [8,41,42]. Our current findings, together with the recent reports showing that the combination of NSAIDs with ω-3 PUFAs used for anti-inflammatory synergism offer protection against gastric damage induced by COX inhibitors [43,44], provide strong evidence of the benefit that could be accrued by combining the two compounds. Besides, such combination could lead to dose reduction of both agents, which could address their potential adverse effects when used alone at higher doses.

In our previous work, we showed that DHA and other PUFAs were very effective at inducing apoptosis and proposed that the elevated levels of COX-2 in various cancers may convert the more effective inhibitory PUFAs into ineffective prostanoids with significantly diminished abilities to control cell proliferation [26,39]. A logical explanation to this phenomenon is that overexpression of COX-2 in tumors likely depletes COX-2 substrates (PUFAs), which have been reported to exhibit tumor suppressive effects. However, several studies have reported that COX inhibitors such as NSAIDs and selective COX-2 inhibitors exert significant antiproliferative effects via multiple mechanisms [39,42,45]. Indeed, treatment with either DHA or NSAIDs alone inhibited the various cancer cell lines in a concentration-dependent manner, an effect that was enhanced by combining the two compounds at relatively low concentrations. Diclofenac appears to be the most effective in enhancing the inhibitory effect of DHA on cell viability. Evidently, co-treatment with DHA and diclofenac exert marked synergistic inhibitory effects on both H1573 and A549 cell viability. A possible reason for the synergistic effect exhibited by diclofenac with DHA may be related to diclofenac’s potent inhibitory effect against COX-2 isoform commonly overexpressed in most tumors. Diclofenac, a phenylacetic acid derivative, which inhibits the COX-2 enzyme with greater potency than COX-1 compared to the other NSAIDs [35]. Thus, the current observation is consistent with previous reports that NSAIDs and COX-2 selective inhibitors (such as celecoxib), which are potent enough to inhibit COX-2 effectively, induce cytotoxicity in various cancer cell lines [39,45,46,47]. Further analysis indicated that the inhibitory effect of DHA and diclofenac treatment on cell viability was triggered via apoptotic cell death. The anticancer activity of the co-treatment was associated with caspase activation, repression of antiapoptotic proteins, such as Bcl-2, Bcl-XL, and Mcl-1, and promotion of proapoptotic proteins, such as Bax and Bim.

In our study, we observed a decreased in expression of K-Ras, N-Ras, and H-Ras proteins as well as reduced levels of activated pan-Ras, K-Ras, p-p44/42, and Akt by co-treatment with DHA and diclofenac. Members of the Ras family of GTPases have been implicated in lung carcinogenesis and are known to mediate signaling pathways that regulate proliferation, survival, and metastasis in cancer cells [38,48,49,50]. KRAS is the most frequently mutated isoform in lung cancer representing 19% of the cases, followed by NRAS (1%) and HRAS (< 1%). The G12 hotspot mutations comprise 83% of all KRAS mutations, and the A549 and NCI-H1573 cell lines used in this study both harbor KRAS mutations at G12 (KRAS-G12S and KRAS-G12A, respectively) [51]. Compounds that prevent post-translational modification of Ras and its plasma membrane targeting are likely to inhibit oncogenic Ras signaling [47,49]. Previous reports from our lab and others suggest that PUFAs alter the plasma membrane binding domains of Ras to disrupt signaling [26,39,40,52,53]. Since Ras proteins are central to several signaling cascades, downregulation of the RAS genes observed in the gene expression study and suppressing expression of all the Ras isoforms studied in the western blot analysis, are expected to impact critical oncogenic pathways. Subsequently, co-treatment of lung cancer cells with DHA and diclofenac also altered the expression of genes involved in these key pathways.

Activated Ras proteins exploit several downstream effectors including activation of the Raf/MAPK pathway and the PI3K/Akt pathway to promote cell survival [38]. To further examine the influence of DHA and diclofenac on these critical signaling pathways, p42/44 ERK activation and Akt activation were determined by immunoblotting. Collectively, our data indicate that DHA and diclofenac modulate Ras-dependent signal transduction by inhibiting activation of both Akt and p42/44 ERK. This is significant because inhibition of pERK is reported to markedly increased pAKT levels, and blocking the PI3K pathway leads to increased activity in the MAPK/ERK pathway via a feedback loop, which ensures that the signal for cellular survival is transmitted downstream [19]. Thus, dual inhibition of Akt and pERK is beneficial since preclinical studies in several tumor types have shown that dual inhibition of both the PI3K and MEK/ERK pathways leads to greater growth inhibition compared to single pathway inhibition [19]. Inactivation of p42/44 ERK disrupts the MAP kinase signaling pathway, preventing phosphorylation of vital cytoplasmic substrates and nuclear transcription factors, as well as other kinases. Alteration of several genes in the PI3K/Akt pathway by DHA and co-treatment with diclofenac is consistent with the recent reports that both compounds inhibit cancer growth and development by inactivation of the PI3K/Akt pathway [15,27,30].

Tumor relapse continues to pose a major concern in the management of lung cancers. An estimated 19.3% to 75% of NSCLC patients experience recurrence after complete surgical resection [54]. Our results indicate that combination treatment with DHA and diclofenac diminishes both anchorage-dependent and anchorage-independent formation and survival of colonies. Consistent with this result is the observed downregulation of proteins involved in cell survival and anti-apoptotic genes as well as genes promoting cell proliferation (Table S1), justifying the potential of combined DHA and diclofenac to prevent tumor relapse.

In summary, the results of the present study indicate that co-treatment with DHA and diclofenac markedly inhibits cell growth and survival of cancer cells and might be a promising strategy for lung cancer chemoprevention as well as adjuvant therapy. This combination therapy may synergize the anticancer effects of these drugs and provide several advantages including better efficacy, prevention of drug resistance resulting from clonogenic resistance, as well as dose reduction of the individual agents involved to minimize their adverse drug reactions. While these benefits appear attractive, further exploration is required to establish the safety profile of this combination in whole animal studies, which is the next step in our research.

## 4. Materials and Methods

### 4.1. Materials and Reagents

Docosahexaenoic acid, and diclofenac were obtained from Cayman Chemical (Ann Arbor, MI, USA). Indomethacin, naproxen, piroxicam, resazurin, horseradish peroxidase-labeled mouse anti-β-actin, anti-K-Ras, anti-N-Ras, and anti-H-Ras antibodies were purchased from Sigma-Aldrich Chemical Co. (St. Louis, MO, USA). Antibodies specific for Bax, Bim, Mcl-1, Bcl-xL, PARP, cleaved PARP, pro-caspase 3, pro-caspase 7, cleaved-caspase 3, phospho-MEK1/2-Ser217/221, phospho-p44/42 MAPK (Erk1/2)-Thr202/Tyr204, phospho-p90RSK-Ser380, phospho-Akt (Ser473), Akt (pan), horseradish peroxidase-labeled anti-mouse, and anti-rabbit immunoglobulins were obtained from Cell Signaling Technology (Beverly, MA, USA). The Ras Activation Assay Kit was purchased from Cytoskeleton Inc. (Denver, CO, USA).

### 4.2. Cell Lines

Human lung cancer NCI-H1573, A549, NCI-H1299, and NCI-H1975 cells were purchased from American Type Culture Collection (ATCC, Manassas, VA, USA). A549 cells were routinely cultured in F12 Kaighn’s medium (Invitrogen, Carlsbad, CA, USA), and NCI-H1573, NCI-H1299, and NCI-H1975 were cultured in RPMI 1640 medium (Invitrogen, Carlsbad, CA, USA). The media used for the routine cell culture were supplemented with 10% (*v/v*) fetal bovine serum (FBS) and a 1% (*w/v*) combination of penicillin, streptomycin, and neomycin (Invitrogen, Carlsbad, CA, USA). The cultures were incubated in 75 cm^2^ vented culture flasks at 37 °C in 5% CO_2_/95% humidified air. The cells were trypsinized after they were 80–90% confluence and seeded onto the appropriate well plates.

### 4.3. Cell Viability Assay

Cells were seeded at a density of 1 × 10^4^ per well in 96-well tissue culture plates and allowed to attach overnight at 37 °C in 5% CO_2_/95% humidified air. The cells were then treated with DHA (0–50 μM) alone or with diclofenac (DCF), piroxicam, or indomethacin (0–100 μM). Identical concentrations of the compounds were used to supplement the samples at 24 h and 48 h, followed by the resazurin reduction method to determine cell viability. After our initial assessment of cell viability at 24 h, 48 h and 72 h, we choose the 48-h time point for further assays because it yielded more consistent results. Cell viabilities were expressed as the percentage of the fluorescence in the treated cells relative to that of the controls. In another experiment, to establish the concentration of diclofenac required for further assays, A549 cells were then treated with diclofenac (0–500 μM) alone or with DHA (0–10 μM). The data for NCI-H1573 and A549 cells, which showed prominent susceptibility to treatment, were analyzed by CompuSyn software. The results were shown in the combination index (CI) values, where CI value < 1, = 1, and > 1 refer to synergistic, additive, and antagonistic effects, respectively.

### 4.4. Cell Apoptosis Assays

The mechanism by which co-treatment with DHA and diclofenac-induced cell death was assessed using AO/EB staining, annexin V/Propidium iodide flow cytometry, and caspase 3/7 activation assays.

#### 4.4.1. Acridine Orange/Ethidium Bromide (AO/EB) Double Staining Assay

A double staining assay with AO/EB was employed to quantify apoptosis in A549 cells after exposure to DHA (0–10 µM) alone or with diclofenac (25 μM) for 48 h [55]. Cells were harvested, washed with PBS, and the cell pellets were suspended in 25 μL of cold PBS and stained with 2 μL of the AO/EB solution (100 μg/mL). The fluorescent morphological changes of the cells were determined with a fluorescent microscope (DM5000B, Leica, Buffalo Grove, IL, USA) fitted with a digital camera (DFC 480, Leica) at 20× magnification.

#### 4.4.2. Annexin-V-FITC Assay

For the flow cytometry analysis, A549 cells seeded at 1 × 10^5^ cell per mL in 6-well plates were treated with DHA (0–10 µM) alone or with diclofenac (25 μM) for 48 h. Apoptosis was determined using the ApopNexin FITC Apoptosis Detection Kit (EMD Millipore, CA, USA) according to the manufacturer’s protocol. Briefly, treated cells were harvested and washed twice with cold PBS and suspended in binding buffer. Annexin-V-FITC and propidium iodide were added to the cell suspensions and incubated for 15 min at room temperature in the dark. The analysis was done by flow cytometry using CytoFLEX (Beckman Coulter, CA, USA).

#### 4.4.3. Caspase-3/7 in situ Assay

Caspase activation was determined using the CaspaTag Caspase-3/7 in situ Assay Kit, Fluorescein (EMD Millipore, Temecula CA, USA) according to the manufacturer’s instructions. A549 cells were treated with DHA (0–10 µM) alone or with diclofenac (25 μM) for 48 h. Cells were stained with 30×FLICA reagent and Hoechst solution (0.5%) followed by washing and addition of fixative. Fluorescent images were obtained with Cytation 1 Cell Imaging Multi-Mode Reader (BioTek, Winooski, VT, USA) at 10× magnification.

### 4.5. Clonogenic Cell Survival and Anchorage-Independent Growth Assays

Cultured NCI-H1573 and A549 cells were seeded at 2.0 × 10^5^ cells per well in 6-well culture plates and incubated overnight at 37 °C in 5% CO_2_/95% humidified air. The cells were treated with DHA (0 –10 µM) alone or with diclofenac (25 μM) for 48 h after which the plates were washed, trypsinized, and counted. In another experiment, the cells were exposed to diclofenac (0–50 µM) alone or with DHA (5 μM) for 48 h. The cells were then plated at 1000 cells/well and incubated in fresh medium containing 10% (*v/v*) fetal bovine serum for an additional 10–14 days. The resulting colonies were fixed with a 10:1 (*v/v*) mixture of methanol and acetic acid, stained with 1% crystal violet, and the number of colonies containing > 50 cells were counted.

Anchorage-independent growth was assayed based on growth in soft agar prepared in medium containing serum and 0.5% agarose (Sigma, St. Louis, MO, USA) in 6-well culture plates and overlayered with 5000 cells resuspended in medium containing serum and 0.33% agarose (Sigma, St. Louis, MO, USA). Cells were incubated at 37 °C for 3 weeks. Resulting colonies were stained overnight with the MTT (Sigma, St. Louis, MO, USA) and the number of colonies containing > 7 µm in diameter were estimated using ImageJ (https://imagej.nih.gov/ij/).

### 4.6. Effects of DHA with Diclofenac on the Expression of Cancer-Related Genes

To determine which pathways were affected by the combination treatments, lung cancer A549 and NCI-H1573 cells were seeded at a density of 1 × 10^5^ per well in 12-well culture plates and allowed to attach overnight at 37 °C in 5% CO_2_/95% humidified air. The cells were then treated with either solvent (vehicle control) or DHA (5 μM and 10 μM) alone or with diclofenac (25 μM). After 48 h of treatment, the cells were detached with trypsin, washed once with PBS, counted, and 5000 cells were lysed in 5 μL RLT buffer (Qiagen, Valencia, CA, USA). The analysis for the relative expression of cancer-related genes was assessed using the nCounter PanCancer Pathways Panel, which targets 770 genes from 13 canonical pathways (NanoString Technologies, Seattle, WA, USA). Expression data were normalized using the nSolver 3.0 analysis Software (NanoString Technologies, Seattle, WA, USA) analysis module and custom scripts in R 2.13.1. Background correction was done by subtracting the geometric mean of the eight negative-control probes. Expression values were normalized with the most stable 31 housekeeping genes, which were selected based on the geNorm algorithm. Expression values were then log_2_-transformed and standardized within each sample. Multi Experiment Viewer (MeV v4.6.2) was used to cluster the data sets to obtain the heat maps resulting from the numerical fold-change expression levels compared to the control. Before clustering, the data was filtered using criteria set such that only data values ≥ ± 2-fold changes were employed. A total of 60 genes common to both cell lines passed the filtering criteria were further grouped according to their associated signaling pathways, as described by the nCounter PanCancer Pathways code set. Genes associated with Ras/MEK/ERK and PI3K/AKT were used for the heat map analyses. Color scale limits were set at “−3.0, 0.0, 3.0”, meaning that the brightest red represents ≥ 3-fold upregulation relative to the controls, the brightest green represents ≥ 3-fold downregulation, and black represents no change.

### 4.7. Immunoblotting

Cultured cells were seeded at 2 × 10^5^ cells per well in 6-well culture plates overnight at 37 °C in 5% CO_2_/95% humidified air. The cells were treated with DHA (0–10 μM) alone or with diclofenac (25 μM) for 48 h. The cells were lysed in ice-cold RIPA buffer (10 mM Tris-HCl, 150 mM NaCl, 1% sodium deoxycholate, 1% Triton X-100, and 0.1% SDS) containing protease inhibitors, centrifuged at 10,000 × g for 5 min, and the supernatant was saved. Protein in the lysate was quantitated using the BCA Protein Quantification Kit (Thermo Fisher Scientific, MA, USA), according to the manufacturer’s protocol. Protein samples were run on precast 4 to 20% gradient Tris-HCl gels (Bio-Rad, CA, USA) and then transferred onto polyvinylidene difluoride (PVDF) membranes (Bio-Rad, CA, USA). The membranes were then blocked with 5% non-fat dried milk in Tris-buffered saline (TBS) and Tween 20 (50 mM Tris-HCl pH 7.5, 150 mM NaCl, and 0.1% (*v/v*) Tween 20) for 1 h at room temperature and incubated with the appropriate specific primary antibody overnight at 4 °C. After multiple TBST washes, membranes were incubated with the corresponding horseradish peroxidase-conjugated secondary antibody for 1 h at room temperature. Blots were then visualized by enhanced chemiluminescence using the ChemiDoc™ XRS+ Imaging System (Bio-Rad, CA, USA).

#### RAS Pull-Down Assay

Pull-down assay was performed using a glutathione S-transferase fusion protein corresponding to the human Ras-binding domain of Raf-1, which specifically binds to the GTP-bound form of Ras (Cytoskeleton Inc., CO, USA). A549 cells were treated with DHA (5 and 10 μM) alone or with DCF (25 μM) for 48 h. Epidermal growth factor (100 ng/mL) was added after 48 h of treatment and the cells were lysed 15 min later. Equal amounts of protein lysates were incubated with agarose beads coated with Raf1 Ras-binding domain and active Ras was then analyzed by immunoblotting using anti-pan-Ras and anti-K-Ras antibodies. Aliquots of total lysates containing 30 μg of protein each were used to assess total Ras protein.

### 4.8. Statistical Analysis

All results were expressed as the means ± S.E.M. The concentration-response curves were obtained by plotting the percentage inhibition against the log of the inhibitor concentrations. Nonlinear regression plots were generated using GraphPad Prism version 5.0 for Windows (San Diego, CA, USA). From these, the concentrations that inhibit 50% of the activity (IC_50_) were calculated. Statistical significance was determined by either one-way ANOVA with Dunnett’s post-hoc test or by Student’s *t*-test as indicated in figure legends. For all the statistical analysis: * = *p* ≤ 0.05, ** = *p* ≤ 0.01, *** = *p* ≤ 0.001.

## 5. Conclusions

The data from the current study demonstrate enhanced anti-cancer potential by combining low doses of diclofenac with DHA, which can be further developed for chemopreventive/adjunct therapeutic use. A safe and efficacious combination therapy of diclofenac and DHA can serve as an adjunct to surgery and prevent the formation of new lesions, while lowering the overall risk of disease progression.

## Figures and Tables

**Table 1:** IC_50_ of DHA alone and co-treatment with non-steroidal anti-inflammatory drugs (25, 50 and 100 µM) in NCI-H1573, A549, NCI-H1299, and NCI-H1975 human lung cancer cells.

Cell Line	IC_50_ of DHA (µM)				
	NSAID	DHA Alone	DHA + NSAID		
			25 µM	50 µM	100 µM
**NCI-H1573**	Diclofenac	9.5 ± 1.3	4.5 ± 0.4	3.2 ± 0.4	3.0 ± 0.3
	Piroxicam	9.5 ± 0.6	~9.8	10.1 ± 1.3	8.3 ± 0.2
	Indomethacin	~10.3	7.0 ± 0.2	7.0 ± 0.3	6.1 ± 0.1
	Naproxen	~10.0	~9.8	11.3 ± 0.4	10.0 ± 0.3
**A549**	Diclofenac	9.5 ± 1.1	4.3 ± 0.3	4.0 ± 0.3	3.7 ± 0.4
	Piroxicam	9.0 ± 1.5	4.9 ± 1.1	5.0 ± 0.6	5.3 ± 0.7
	Indomethacin	10.3 ± 0.1	5.8 ± 0.1	5.7 ± 0.0	5.5 ± 0.1
	Naproxen	9.4 ± 0.8	5.9 ± 0.6	7.3 ± 0.1	6.4 ± 0.2
**NCI-H1299**	Diclofenac	9.8 ± 0.3	10.4 ± 0.4	11.3 ± 0.2	8.5 ± 0.5
	Piroxicam	10.2 ± 0.5	9.7 ± 0.2	8.7 ± 0.4	8.4 ± 0.1
	Indomethacin	9.1 ± 0.1	5.9 ± 0.0	4.9 ± 0.3	3.1 ± 0.3
**NCI-H1975**	Diclofenac	~11.2	~11.2	7.3 ± 0.4	5.8 ± 0.3
	Piroxicam	10.0 ± 1.0	~10.0	8.8 ± 0.1	8.2 ± 0.2
	Indomethacin	11.9 ± 0.8	9.7 ± 0.6	9.2 ± 0.1	8.0 ± 0.2
	Naproxen	9.8 ± 0.2	8.1 ± 0.3	9.6 ± 0.3	9.2 ± 0.3

**Table 2:** IC_50_ of diclofenac alone and co-treatment with DHA (2, 5 and 10 µM) in NCI-H1573 and A549 human lung cancer cells.

Cell Line	IC_50_ of Diclofenac (µM)			
	Diclofenac Alone	Diclofenac + DHA		
		2 µM	5 µM	10 µM
NCI-H1573	87.5 ± 9.6	71.4 ± 9.0	48.3 ± 1.4	25.0 ± 3.4
A549	92.8 ± 9.9	83.1 ± 4.3	73.5± 6.5	11.3 ± 3.5

## Supplementary Materials

The following are available online at https://www.mdpi.com/2072-6694/12/9/2683/s1, Figure S1: Detailed information about western blot in Figure 7, Figure S2: Detailed information about western blot in Figure 8, Table S1: The major pathways and genes whose levels of expression were significantly altered in A549 and H1573 cancer cell lines treated with the indicated concentrations of DHA and diclofenac.

## Abbreviations

DHA	docosahexaenoic acid
EPA	eicosapentaenoic acid
COX	cyclooxygenase
NSCLC	non-small cell lung cancer
NSAIDs	non-steroidal anti-inflammatory drugs
DCF	diclofenac
PUFAs	polyunsaturated fatty acids
PGs	prostaglandins
CI	combination index
GTPase	nucleotide guanosine triphosphate hydrolase
NFκB	nuclear factor kappa-light-chain-enhancer of activated B
TGF-β	transforming growth factor beta
MEK	mitogen-activated protein kinase/extracellular signal-regulated kinase
ERK	extracellular signal-regulated kinase
PPARs	peroxisome proliferator-activated receptors
MAPK	mitogen-activated protein kinase
PI3K	phosphoinositide 3-kinase
Akt	protein kinase B
mTOR	mammalian target of rapamycin
JAK	janus kinase
STAT	signal transducer and activator of transcription
FITC	fluorescein isothiocyanate
RIPA	radioimmunoprecipitation assay
BCA	bicinchoninic acid assay
GADD45A	growth arrest and DNA damage-inducible alpha
TNFAIP3	tumor necrosis factor alpha-induced protein 3
CDC25C	M-phase inducer phosphatase 3
E2F1	transcription factor
PCNA	proliferating cell nuclear antigen
CCNA2	cyclin-A2
CCNB1	G2/mitotic-specific cyclin-B1
CCNE2	cyclin E2
HDAC2	histone deacetylase 2
POLE2	DNA polymerase epsilon subunit 2
UBE2T	ubiquitin-conjugating enzyme
HIST1H3B	histone H3.1
CDKN2C	cyclin-dependent kinase 4 inhibitor C
