# Supplementary material for: Diclofenac Enhances Docosahexaenoic Acid-Induced Apoptosis in Vitro in Lung Cancer Cells
Source: Cancers (Basel). 2020 Sep 20;12(9):2683. doi: 10.3390/cancers12092683 (PMC7564004; doi:10.3390/cancers12092683)
Supplement: Supplementary file 1 [file cancers-12-02683-s001.pdf]

Supplementary Materials

Diclofenac Enhances Docosahexaenoic Acid-Induced Apoptosis in Lung Cancer Cells

Rosemary A. Poku, Kylee J. Jones, Megan Van Baren, Jamie K. Alan and Felix Amissah

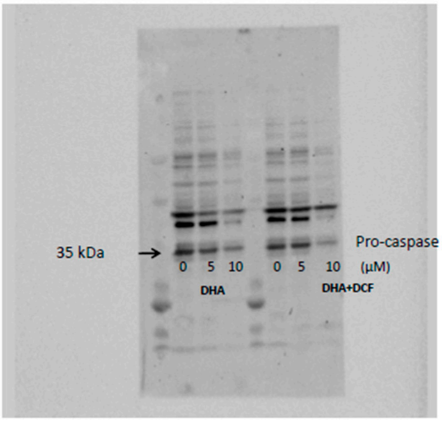

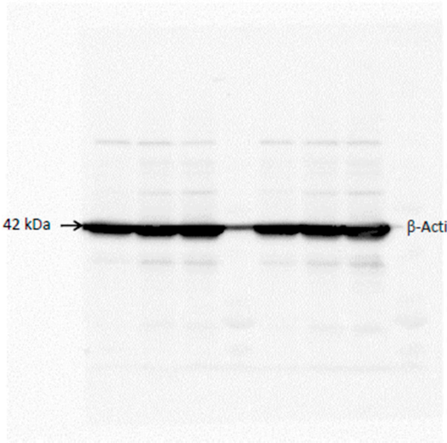

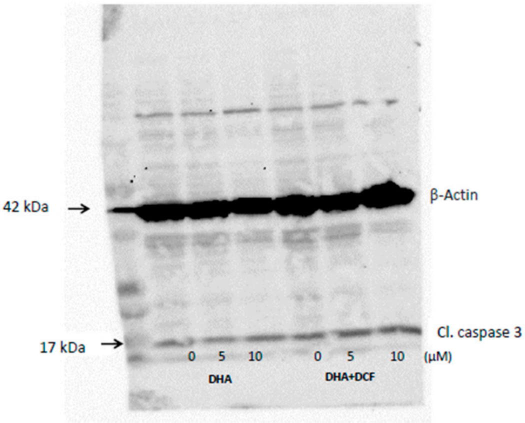

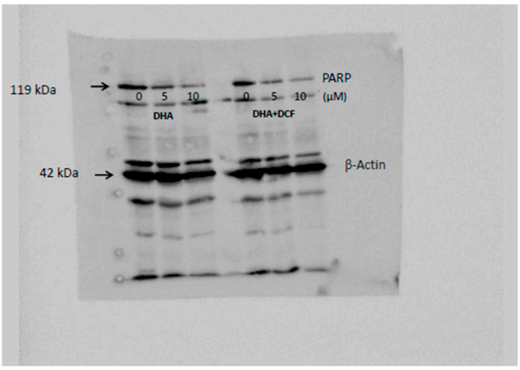

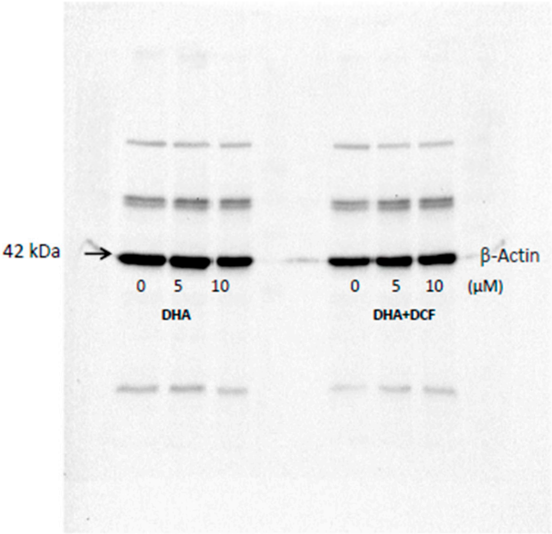

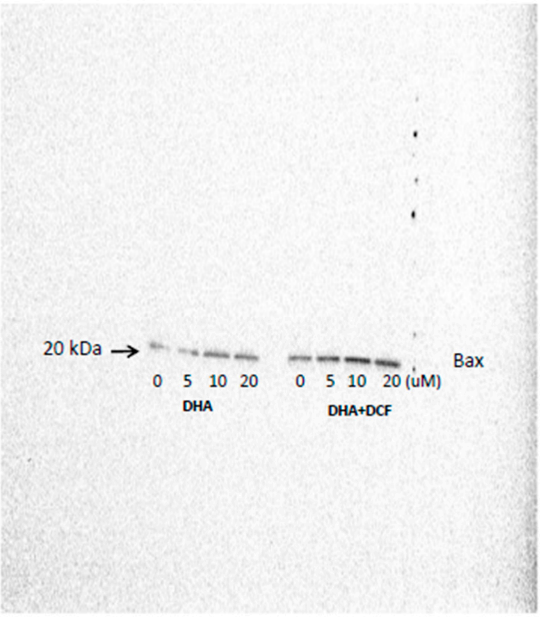

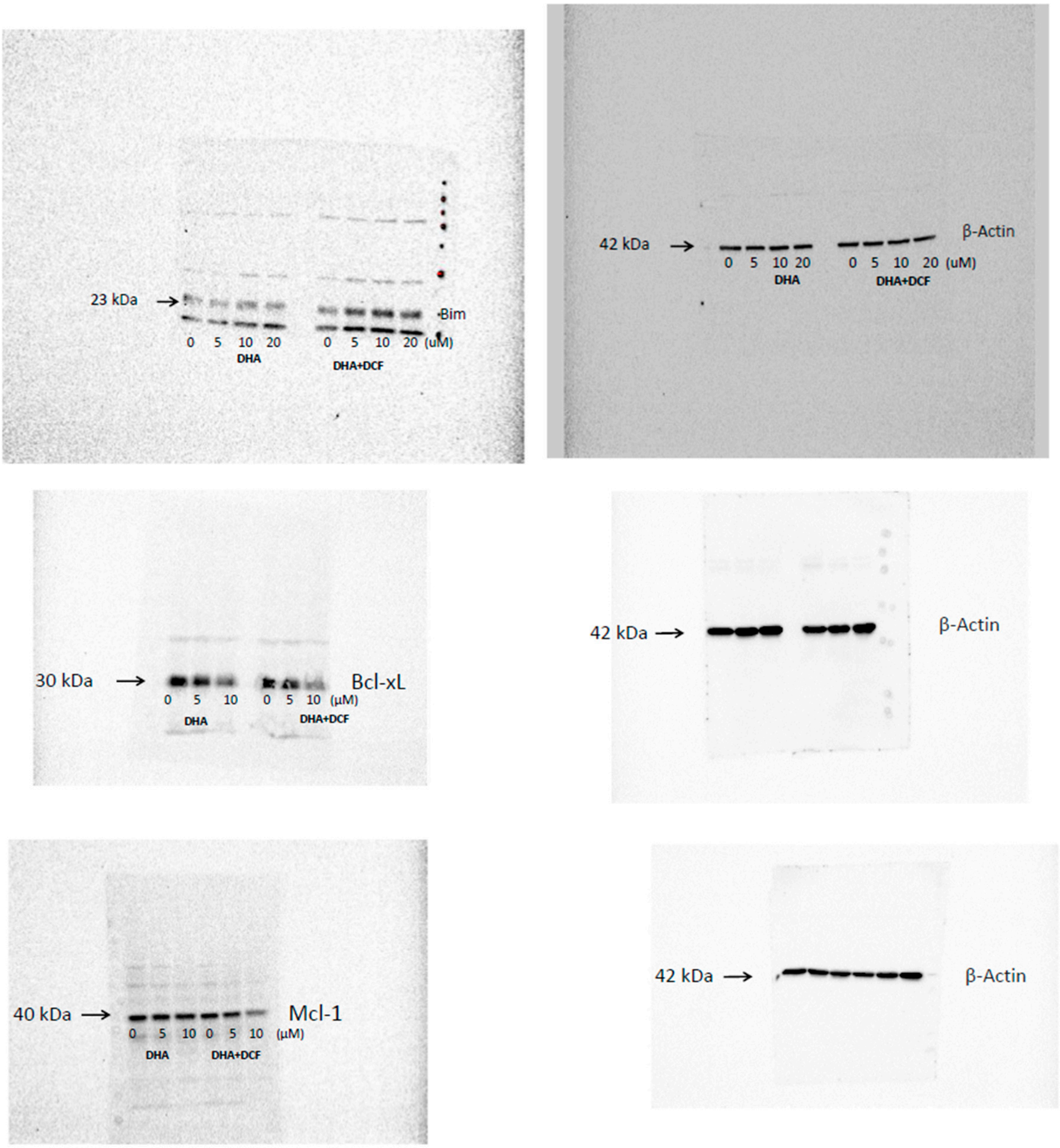

Figure S1. Detailed information about western blot in Figure 7.

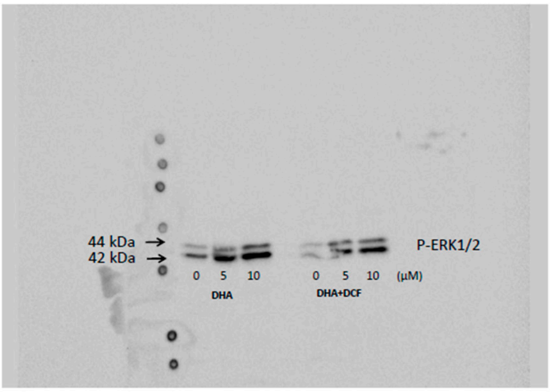
44 kDa
42 kDa
P-ERK1/2
0 5 10 0 5 10 (μM)
DHA
DHA+DCF

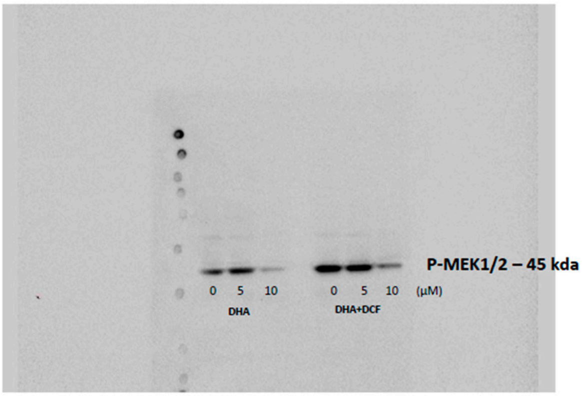
P-MEK1/2 – 45 kda
0 5 10 0 5 10 (μM)
DHA
DHA+DCF

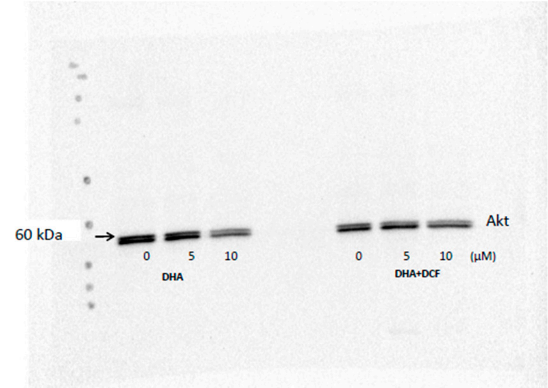
60 kDa
Akt
0 5 10 0 5 10 (μM)
DHA
DHA+DCF

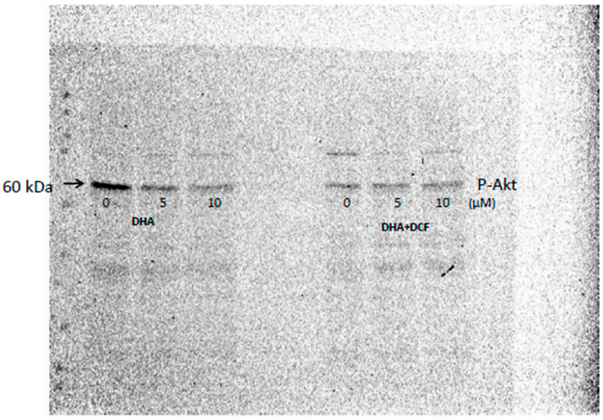
60 kDa
P-Akt
0 5 10 0 5 10 (μM)
DHA
DHA+DCF

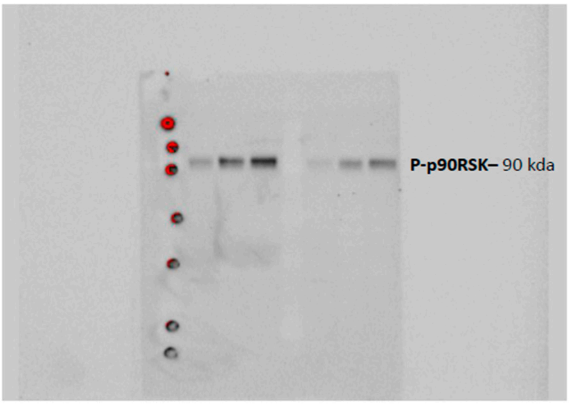
P-p90RSK– 90 kda

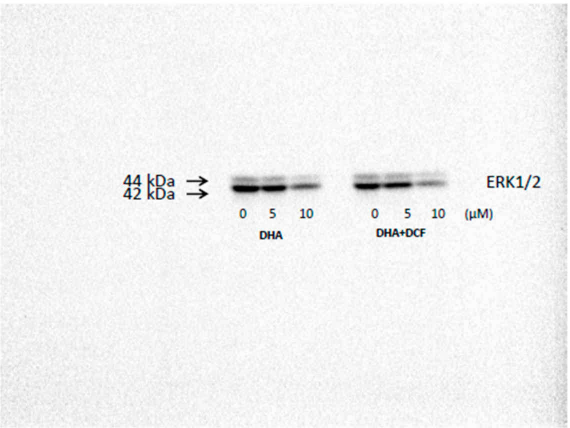
44 kDa
42 kDa
ERK1/2
0 5 10 0 5 10 (μM)
DHA
DHA+DCF

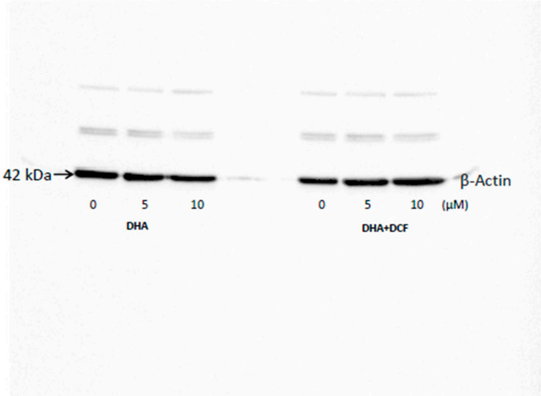
42 kDa
β-Actin
0 5 10 0 5 10 (μM)
DHA
DHA+DCF

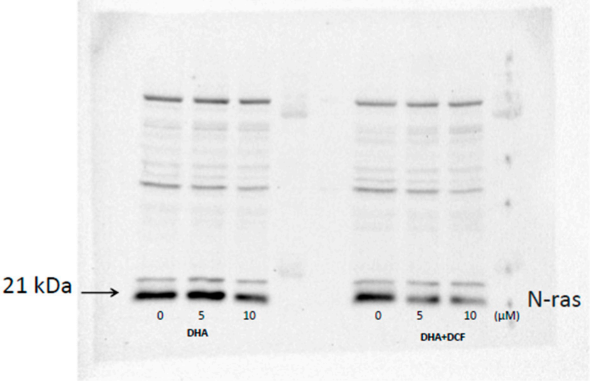
21 kDa
N-ras
0 5 10 0 5 10 (μM)
DHA
DHA+DCF

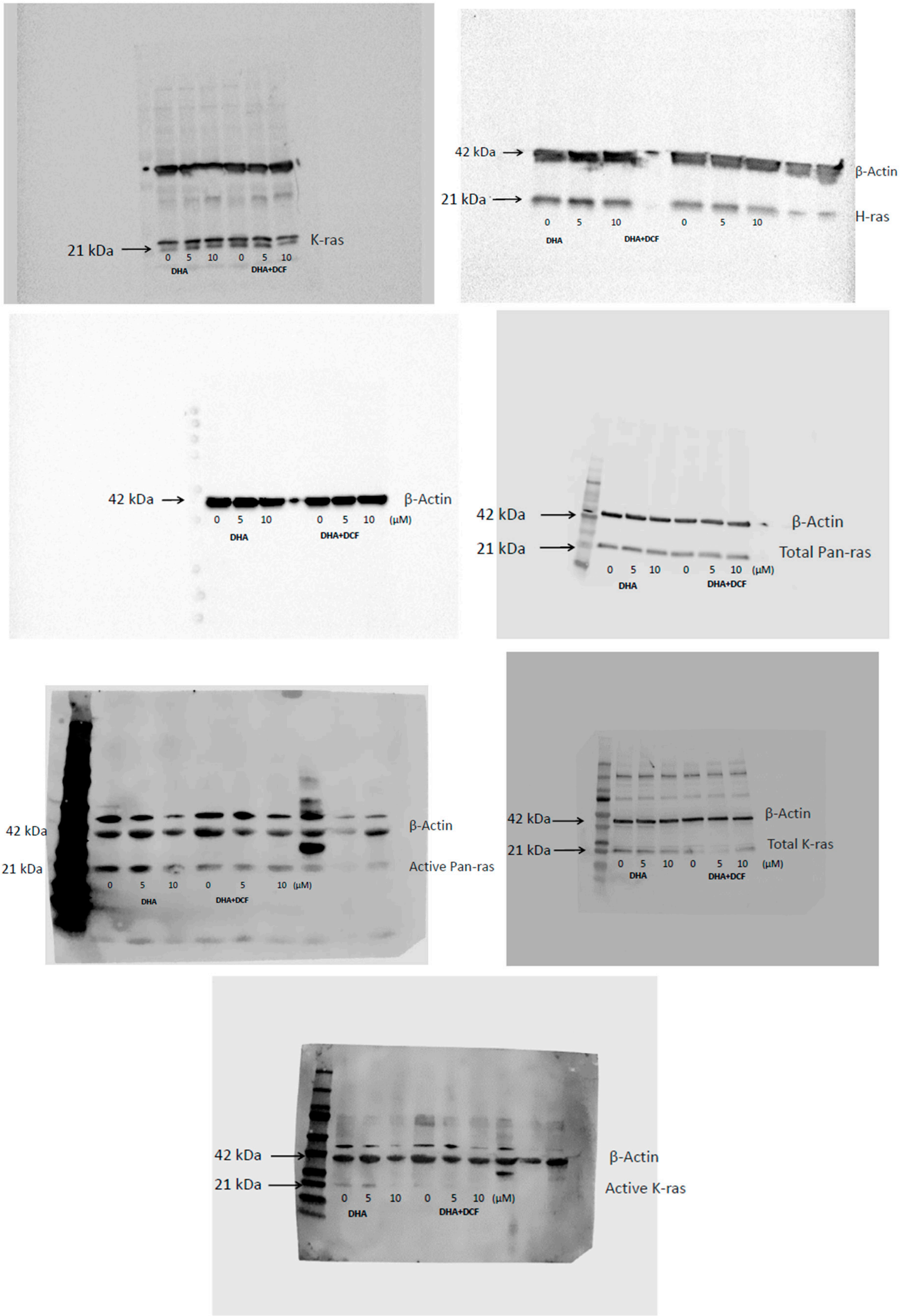

Figure S2. Detailed information about western blot in Figure 8.

Table S1. The major pathways and genes whose levels of expression were significantly altered in A549 and H1573 cancer cell lines treated with the indicated concentrations of DHA and diclofenac.

Probe Name	Fold Change									
	A549					H1573				
	DHA (5 µM)	DHA (10 µM)	DCF (25 µM)	DCF (25 µM) +DHA (5 µM)	DCF (25 µM) +DHA (10 µM)	DHA (5 µM)	DHA (10 µM)	DCF (25 µM)	DCF (25 µM) +DHA (5 µM)	DCF (25 µM) +DHA (10 µM)
**MAPK Signaling Pathway**										
CACNA1D	−2	1	2	2	2	−1	−1	−3	−4	−4
GADD45A	−1	1	2	2	3	−1	−1	3	4	6
NR4A1	−2	−2	−2	−2	−2	1	−2	−4	−4	−7
NTF3	−1	−1	−1	−5	−2	1	1	−8	−15	−15
PDGFC	−2	−1	2	1	3	−2	3	1	2	2
PLA2G4A	−1	−1	−1	−1	−2	3	−2	−1	−4	−5
RAC1	1	−1	−2	−1	−5	−1	−2	2	−4	−4
TP53	−1	−1	−2	−1	−2	2	−2	1	−3	−3
IKBKG	2	2	3	3	2	−1	2	1	2	3
KRAS	−1	−1	1	1	−2	1	−1	−2	−2	−2
NRAS	−1	−1	1	1	−2	1	2	1	−2	−2
HRAS	−2	−2	−2	−2	−2	2	2	2	−1	−2
**PI3K-AKT Signaling Pathway**										
BRCA1	−1	−1	−2	−2	−2	−3	−1	−1	−2	−2
CCND3	−2	−2	−2	−4	−3	−1	2	−3	−3	−2
CCNE2	−2	−2	−3	−4	−4	1	−2	−3	−9	−5
COL24A1	−3	−2	−1	−1	−2	1	−2	−8	−11	−5
DDIT4	−2	−1	2	1	3	−2	2	3	3	4
ITGA2	1	2	3	3	4	−1	2	−1	1	2
LAMC2	−1	1	2	2	4	2	4	2	3	5
NR4A1	−2	−2	−2	−2	−2	1	−2	−4	−4	−7
PDGFC	−2	−1	2	1	3	−2	3	1	2	2
PIK3CD	−2	−1	2	1	3	2	1	2	2	3
RAC1	1	−1	−2	−1	−5	−1	−2	2	−4	−4
TP53	−1	−1	−2	−1	−2	2	−2	1	−3	−3
CREB3L4	−3	−2	−3	−2	−3	−2	−1	−2	−3	−2
IKBKG	2	2	3	3	2	−1	2	1	2	3
KRAS	−1	−1	1	1	−2	1	−1	−2	−2	−2
NRAS	−1	−1	1	1	−2	1	2	1	−2	−2

HRAS	−2	−2	−2	−2	−2	2	2	2	−1	−2
**Cell Cycle/Apoptosis**										
CCNA2	−2	−2	−3	−4	−6	1	−1	−2	−4	−4
CCNB1	−1	−2	−2	−3	−4	1	−1	−2	−4	−4
CCND3	−2	−2	−2	−4	−3	−1	2	−3	−3	−2
CCNE2	−2	−2	−3	−4	−4	1	−2	−3	−9	−5
CDC25C	−2	−2	−2	−3	−3	1	2	−2	−3	−3
CDKN2A	−2	−2	−1	−2	−2	−2	1	−3	−5	−2
CDKN2C	−2	−2	−2	−4	−4	1	−1	−3	−7	−5
CHEK1	−1	−1	−2	−2	−2	1	−2	−1	−2	−2
E2F1	−1	−1	−3	−3	−3	−1	−1	−2	−4	−5
GADD45A	−1	1	2	2	3	−1	−1	3	4	6
HDAC2	1	−1	−2	−1	−2	3	−1	2	−2	−2
IKBKG	2	2	3	3	2	−1	2	1	2	3
IRAK2	1	2	2	2	3	5	1	2	2	−3
MCM4	−2	−2	−3	−4	−4	−1	−1	−2	−3	−5
MCM5	−2	−2	−3	−5	−3	1	−1	−3	−3	−6
PCNA	−2	−2	−2	−4	−5	−3	−4	−3	−12	3
PIK3CD	−2	−1	2	1	3	2	1	2	2	−2
PTTG2	−2	−2	−2	−4	−3	−1	1	−3	−4	2
TNFRSF10A	−1	1	2	1	3	1	2	1	2	4
TNFRSF10B	−1	1	2	1	3	−1	3	2	3	−3
TP53	−1	−1	−2	−1	−2	2	−2	1	−3	−3
TTK	−1	−1	−2	−3	−3	−1	−1	−1	−3	3
**Driver Genes**										
BRCA1	−1	−1	−2	−2	−2	−3	−1	−1	−2	−2
BRCA2	−1	−1	−2	−2	−2	−1	1	−2	−2	−2
CDKN2A	−2	−2	−1	−2	−2	−2	1	−3	−5	−2
HIST1H3B	−1	−2	−4	−5	−9	1	−2	−3	−11	−13
TP53	−1	−1	−2	−1	−2	2	−2	1	−3	−3
RET	−5	−2	−15	−3	−6	2	1	−2	−4	−2
KRAS	−1	−1	1	1	−2	1	−1	−2	−2	−2
NRAS	−1	−1	1	1	−2	1	2	1	−2	−2
HRAS	−2	−2	−2	−2	−2	2	2	2	−1	−2
**APC/Wnt Signaling Pathway**										
CCND3	−2	−2	−2	−4	−3	−1	2	−3	−3	−2
FOSL1	−1	1	2	2	4	−1	2	1	3	4
RAC1	1	−1	−2	−1	−5	−1	−2	2	−4	−4
RHOA	−2	−2	−1	−2	−2	−1	2	−2	−3	−2
TP53	−1	−1	−2	−1	−2	2	−2	1	−3	−3

WNT5A	−2	−3	−3	−5	−5	−2	−1	−3	−4	−3
**Ras Signaling Pathway**										
MLLT4	−1	−1	2	1	3	−4	1	−1	2	3
PDGFC	−2	−1	2	1	3	−2	3	1	2	2
PIK3CD	−2	−1	2	1	3	2	1	2	2	3
PLA2G4A	−1	−1	−1	−1	−2	3	−2	−1	−4	−5
RAC1	1	−1	−2	−1	−5	−1	−2	2	−4	−4
RHOA	−2	−2	−1	−2	−2	−1	2	−2	−3	−2
RIN1	−2	−1	2	−1	3	−1	2	−1	2	2
IKBKG	2	2	3	3	2	−1	2	1	2	3
KRAS	−1	−1	1	1	−2	1	−1	−2	−2	−2
NRAS	−1	−1	1	1	−2	1	2	1	−2	−2
HRAS	−2	−2	−2	−2	−2	2	2	2	−1	−2
**Transcriptional Regulation**										
CDKN2C	−2	−2	−2	−4	−4	1	−1	−3	−7	−5
HDAC2	1	−1	−2	−1	−2	3	−1	2	−2	−2
HIST1H3B	−1	−2	−4	−5	−9	1	−2	−3	−11	−13
HIST1H3H	−2	−2	−3	−5	−7	1	1	−5	−12	−7
TP53	−1	−1	−2	−1	−2	2	−2	1	−3	−3
**DNA Damage Control**										
BRCA1	−1	−1	−2	−2	−2	−3	−1	−1	−2	−2
BRCA2	−1	−1	−2	−2	−2	−1	1	−2	−2	−2
BRIP1	1	−1	−2	−2	−2	1	1	−2	−2	−3
FANCA	1	1	−3	−2	−4	−1	1	2	−1	−3
FANCL	−1	−1	−2	−2	−2	2	−2	1	−2	−6
FEN1	1	−1	−4	−4	−6	3	−3	−1	−4	−4
PCNA	−2	−2	−2	−4	−5	−3	−4	−3	−12	−4
POLE2	1	−1	−3	−2	−7	2	−1	−1	−5	−6
RAD51	−1	−2	−2	−3	−3	2	−1	1	−2	−4
UBE2T	−1	−1	−3	−3	−7	2	−2	1	−5	−4

© 2020 by the authors. Licensee MDPI, Basel, Switzerland. This article is an open access article distributed under the terms and conditions of the Creative Commons Attribution (CC BY) license (http://creativecommons.org/licenses/by/4.0/).
